# Antioxidant and Anti-Inflammatory Effects of *Anacardium occidentale* L. and *Anacardium microcarpum* D. Extracts on the Liver of IL-10 Knockout Mice

**DOI:** 10.1155/2020/3054521

**Published:** 2020-12-07

**Authors:** Anderson Barbosa Baptista, Mariáurea M. Sarandy, Reggiani Vilela Gonçalves, Rômulo Dias Novaes, Cláudio Gonçalves da Costa, João Paulo Viana Leite, Maria do Carmo Gouveia Peluzio

**Affiliations:** ^1^Department of Nutrition and Health, Universidade Federal de Viçosa, Viçosa, Minas Gerais 36570-900, Brazil; ^2^Department of General Biology, Universidade Federal de Viçosa, Viçosa, Minas Gerais 36570-900, Brazil; ^3^Department of Animal Biology, Universidade Federal de Viçosa, Minas Gerais 36570-900, Brazil; ^4^Department of Structural Biology, Universidade Federal de Alfenas, Alfenas, Minas Gerais, Brazil; ^5^Specialized Forensic Analysis Center, Forensic Chemistry and Toxicology Laboratory, Palmas, Tocantins, Brazil; ^6^Department of Biochemistry and Molecular Biology, Universidade Federal de Viçosa, Viçosa, Minas Gerais 36570-900, Brazil

## Abstract

**Background:**

The *Anacardium occidentale* L. (cashew) and *Anacardium microcarpum* D. (cajuí) are plants commonly found in Brazil. They present phytochemical compounds with antioxidant and anti-inflammatory action. Therefore, the objective of this study was to analyze the antioxidant and anti-inflammatory activities of ethanolic extracts from leaves of *A. occidentale* and *A. microcarpum* and its effect on the hepatic tissue in experimental knockout models after they received Paracetamol®.

**Methods:**

Ethanol extracts from *A. occidentale* and *A. microcarpum* leaves were prepared. Total phenolics were determined by Folin–Ciocalteau reagent, and flavonoids are based on the complexation reaction with the aluminum metal, forming a colored complex. Fingerprint HPLC was performed to detect phenolic compounds. Knockout IL-10 mice randomly divided into six groups were used and received the following treatments: G1, only water; G2, *A. occidentale* extract; G3, *A. microcarpum* extract; G4, Paracetamol®; G5, Paracetamol® + *A. occidentale* extract (400 mg/kg); G6, Paracetamol® + *A. microcarpum* extract (400 mg/kg). Biochemical parameters of the blood and differential count of leukocytes were done. Oxidative markers and histopathological analyses were performed on their liver tissue.

**Results:**

Phenolic compounds and total flavonoids were detected in both two extracts analyzed. The HPLC fingerprint detected phenolic acid, gallic acid, and catechin flavonoid in the two extracts. Histopathological analyses of the hepatic tissue permitted evaluation of nuclear increase, sinusoid congestion, and inflammatory infiltrate. *A. microcar*pum presented more antioxidant activity increasing antioxidant enzyme levels and reducing TBARS and carbonyl protein when compared to the other treatments after exposure to Paracetamol®. Histopathological analyses showed a decrease in the inflammatory infiltrate after treatment with extracts.

**Conclusion:**

Our findings indicate that both extracts, especially *A. microcarpum*, can reduce hepatic damage in knockout mice exposed to paracetamol, indicating the curative power of these extracts reducing lipid peroxidation and in the morphofunctional damage to the liver parenchyma.

## 1. Introduction

The *Anacardium occidentale* L. (cashew tree) is a plant of the Anacardiaceae family, originating in the northern and northeastern regions of Brazil [1]. The *Anacardium microcarpum* D. (cajuí) is also found in these regions and is widely used in traditional medicine for the treatment of inflammation, rheumatism, tumors, and infectious diseases [2]. *A. occidentale*, also known as the cashew tree, is the most widely cultivated and used species [3]. Both species are rich in phenolic compounds and flavonoids in their leaves, bark, fruits, and nuts. Their high anti-inflammatory and antioxidant power offer protection to the cells [4]. Besides, the leaves, the pseudofruit, and the peel of the *A. occidentale* have polyphenols, mainly tannins, which act as natural antibiotics [5]. The pseudofruit of the *A. microcarpum* contains mainly vitamin C, polyphenols, and minerals and has activity in the prevention of cardiovascular diseases, as well as anti-inflammatory and antimicrobial action [6].

The liver is the main organ of detoxification of the organism, being responsible for biotransformation and elimination of chemical compounds that act in the generation of free radicals. These radicals produce intense lesions in tissues, such as inflammation and degenerative processes that may compromise organ function [7]. Paracetamol® is currently the most widely used analgesic in the world and has recognized hepatotoxic and nephrotoxic effects in animal models. Its harmful effects are related to an increase in the production of reactive oxygen species and the degradation and inhibition of antioxidant enzyme synthesis [8]. The increase of these compounds in hepatic tissues promotes chain reactions characterized by lipid and protein peroxidation [9]. These reactions promote the migration of inflammatory cells and the formation of degenerative lesions that may evolve into irreversible processes such as cell death [10, 11].

Interleukin 10 (IL-10) is an anti-inflammatory cytokine and controls the signaling in macrophages and neutrophils and is necessary to prevent abnormal regulation of responses to the normal inflammatory. In addition, it regulates angiogenesis by inducing the cell-type dependent expression of both angiogenic and angiostatic factors.

Animal models have been used for several years, with a wide variety of species used. Rats and mice are often used due to their small size, high reproduction rate, and several similarities in anatomy, physiology, and genetics when compared to humans, providing important information for the study of some diseases [12, 13]. Animal models of intestinal inflammation have been used for decades and allow a variety of information regarding mucosal immunology, maintenance, and disturbance of intestinal homeostasis, in addition to being able to assess an intestinal inflammation observed in IBDs [14]. The knockout mice for IL-10 (IL-10 -/-) are used in intestinal inflammation disease (DII) studies, as they develop colitis spontaneously and affect the discontinuous ones that affect the entire extension of the small and large intestine. These occurrences occur due to the absence of the cytokine IL-10, which acts in the regulation of intestinal homeostasis. Its deficiency is related to the appearance of inflammatory infiltrates, epithelial hyperplasia, and ulcers, among other changes in the intestinal epithelium [15]. Thus, IL-10 knockout mice are a desirable animal model and once may develop greater inflammation and presented reduced vascularization during the healing process. Currently, it is known that effective tissue repair occurs by the balance of anti- and proinflammatory mediators and proper vascularization. Prolonged inflammation can result in detrimental tissue injury, and a reduced number of blood vessels can slow down the regeneration process [16]. So, how our objective was to study the effect of the *A. occidentale* and *A. microcarpum* on the hepatic tissue after lesion promoted by paracetamol, and we believed that this animal model is very valid because it allows us to study this pathology condition in extreme conditions, with low vascularization and high profile inflammatory. Thus, the use of this animal model enhances the inflammatory process and allows us to have a better understanding of the action of the herbal medicine tested in the hepatic repair process.

In the inflammatory state, the main disorders are related to the imbalance of nitric oxide, lipid peroxidation, activation of cytokine cascades, and mainly the release of reactive oxygen (ROS) and nitrogen (RNS) species, which when overproduced can cause cellular damage; however, the organism uses a defense system capable of minimizing these effects, using various enzymes such as SOD (superoxide dismutase), CAT (catalase) and glutathione peroxidase, and nonenzymatic protein such as carbonylated protein [17].

The anti-inflammatory properties of *Anacardium* extracts need to be better elucidated and the mechanism clarified. Some properties have already been demonstrated in leaves, bark, chestnut, and peduncle and are used in traditional medicine to treat inflammatory diseases such as arthritis. In inflammatory models, the peel extract inhibited the production of prostaglandins by neutrophils and produced anti-inflammatory activity in murine lipopolysaccharide (LPS)-induced microvascular permeability and septic shock assays, the inhibition of inflammatory cytokines, and the expression of the iNOS and COX-2 gene blocking the NF-kB and MAPK pathways and regulators of the inflammatory process [18]. In a study with a Swiss mouse model that used the juice of the peduncle of *A. occidentale* by gavage, to treat cutaneous edema induced experimentally, it was observed that there was a reduction in the inflammatory process, induced by xylene, with reduction of the ear edema and registration of the process healing, compared to control [19]. These effects are attributed to the phenolic compounds present in all parts of *Anacardium* and probably act by altering the release and gene expression of proinflammatory cytokines, such as TNF-*α*, IL-1, and IL-6 [20], and blocking the activities of the 5-lipoxygenase (5-LOX) or cyclooxygenase 2 (Cox-2), inhibiting the biosynthesis of inflammatory mediators [17].

For the liver to perform detoxification of the body, important enzymes must maintain the organ's redox balance. The reduction in oxidative markers and increase in antioxidant enzymes typically indicate the potential for the drag of the free radicals produced in the process of changing the oxidative/antioxidative balance and have been widely used in the study of plant extracts [21, 22]. The discovery and use of new molecules that can reduce the inflammatory and oxidative process are fundamental since the current nonsteroidal drugs can cause side effects, for that the secondary metabolites of vegetables, especially the phenolic ones, are important allies [17, 18]. In addition to the oxidative profile, analysis of some serum enzymes, such as alanine transaminase (ALT), aspartate transaminase (AST), bilirubin (Bb), and gamma-glutamyl phosphatases (GGT), is often used as indirect markers of liver injury and is considered to be sensitive parameters to evaluate the functional status of the organ [23, 24].

Based on this, the objective of the present study was to realize the chemical and biochemical characterization of the extracts and to analyze the antioxidant and anti-inflammatory potential of the leaves *A. occidentale* and *A. microcarpum* on the liver of the knockout animals after exposure to Paracetamol®.

## 2. Materials and Methods

### 2.1. Chemicals

Paracetamol® (composition: 500 mg of paracetamol per ml), Tylenol-Janssen Johnson & Johnson® brand, was used. Thiobarbituric acid, hydrogen peroxide, sodium phosphate, formaldehyde, glutaraldehyde, hematoxylin, and eosin were purchased from Sigma Chemical Co. (St. Louis, MO, USA). Aspartate aminotransferase (AST), alanine aminotransferase (ALT), alkaline phosphatase (ALP), and gamma-glutamyl transferase (GGT) biochemical kits were purchased from Human In Vitro Diagnostics (Minas Gerais, Brazil).

### 2.2. Plant Material

The leaves of *A. occidentale* and *A. microcarpum* were collected from the campus of the Universidade Federal do Tocantins (UFT), Palmas, TO, Brazil. A herbarium voucher specimen was deposited in the ecotonal herbarium (Botanic Teacher DSc. Rodney Haulien Oliveira Viana), at the Tocantins Federal University (numbers 109 and 875, respectively). They were dried and ground in a knife mill. To obtain the ethanolic extract, the previously ground plant material was concentrated in ethanol PA for 72 h, and the supernatant was withdrawn and reserved. This procedure was replicated three consecutive times. The supernatant was filtered, and then the solvent was distilled off in a rotary evaporator at 60°C under reduced pressure. The ethanolic extract was placed in sterile glass vials and in an oven at 40°C to complete the total evaporation of the ethanol [25]. The crude extracts were packed in bottles, wrapped with aluminum foil, and placed in a refrigerator for further analyses. The extraction stage was performed at the Laboratory of Basic Sciences and Health at UFT, Palmas, TO, Brazil.

### 2.3. Chemical Analysis

#### 2.3.1. Quantification of Total Phenolic Compounds

The total phenolic concentration in the ethanolic extract was determined using the Folin–Ciocalteau reagent. Initially, 8.0 mg/mL of the dry extract was diluted in 80% ethanol. 600 *μ*l of the solution was poured into a flask, and 3.0 mL of Folin's reagent was added to it; it was left resting for 3 minutes, after which 2.4 mL of the 4% saturated sodium carbonate solution was added thereto. The tubes rested in the dark for 60 minutes. A white reagent was conducted under the same conditions. A gallic acid calibration curve was prepared at concentrations of 5, 15, 25, 50, 75, 100, 125, 150, 175, and 200 mg/mL. The reading was taken at 760 nm by a UV-vis spectrophotometer. Results were expressed as mg EAG g^−1^ (gallic acid equivalent per gram of dry extract) adapted on Boroski and Perez-Jimenez et al. [26, 27].

#### 2.3.2. Quantification of Total Flavonoid Compounds

The determination of flavonoids is based on the complexation reaction with the aluminum metal, forming a colored complex. The quantity of flavonoids from leaf extracts of *A. occidentale* and *A. microcarpum* was determined by the spectrophotometric method. . For the construction of the calibration curve, the rutin standard was used at concentrations of 10, 20, 40, 60, 80, and 100 mg L^−1^. Solutions containing 1 mg of the extracts of *A. occidentale* and *A. microcarpum* were diluted in 1 mL of methanol. 500 *μ*L of the extract solution was added to 500 *μ*L of 5% aluminum chloride in methanol and supplemented with 2.5 ml of methanol. This mixture was kept protected from light at room temperature for 1 hour and then read at 415 nm on a UV-Vis spectrophotometer against white prepared with 500 *μ*l of aluminum chloride and 2.5 mL of methanol. Results were expressed in mg ER g^−1^ (milligrams rutin equivalent per gram of extract) [28].

#### 2.3.3. High-Performance Liquid Chromatography (HPLC)

The ethanolic extracts, as well as a mixture of authentic samples of phenolic compounds, were analyzed under the same conditions by HPLC (Shimadzu®, Kyoto, Japão) equipped with pump (LC-10AT), degasser (DGU-14A), and UV-vis SPD-10A detector. The column used was the Phenomenex Luna C18 5 *μ*m (250 mm × 4.6 mm) with C18 Phenomenex Security Guard Cartridges (4 × 3.0 mm). Chromatographic elutions were detected at different wavelengths (254 and 280 e 320 nm). The response of the detector was recorded and integrated using the Class-VP software. The mobile phase consisted of 0.1% phosphoric acid in water (phase A) and 0.1% phosphoric acid in water/acetonitrile/methanol (54 : 35 : 11 v/v) (phase B) under the following gradient profile: 0–5 min, 0% B; 5−10 min, 30% B, 10−20 min, 40% B, 20−60 min 40% B, 60–70 min 50% B, 70–90 min 60% B, 90–100 min 80% B, 100–110 min 100% B, and 110–120 min 100% B. The flow rate was 1.0 mL/min. The compounds were identified by comparing the retention times of samples and authentic standards of reference compounds such as gallic acid, chlorogenic acid, vanillic acid, syringic acid, catechin, quercetin, coumaric acid, rutin, naringin, and flavone (Sigma®). For those peaks referring to the patterns of phenolic compounds that appeared with the same retention time in the chromatograms of the extracts, a coinjection of the authentic samples with the extract was performed to confirm the chemical identification. Prior to the analysis, all the extracts (at 1 mg/mL) and authentic standards (0.18 mg/mL) were filtered through 0.20 *μ*m membrane filters of polyvinylidene difluoride. A methodology was based on Araújo et al. and Costa et al. [29, 30].

### 2.4. In Vivo Tests

#### 2.4.1. Experimental Design

Thirty-six 60-day-old male mice, knockout for IL-10, from the Central Vivarium of the Universidade Federal de Alfenas (UFAL), Alfenas, Minas Gerais, Brazil, were used. The animals were kept in collective cages in light/dark cycles of 12 hours and an average temperature of 22 ± 2°C. They received water and commercial feed *ad libitum*. The experimental procedures were approved by the Committee for Ethics in Animal Research Number 23101.001539/2017-08 CEUA-UFT, and the animals were handled according to the norms of the National Council of Control of Animal Experimentation (CONCEA).

The animals were randomly divided into six groups (*n* = 6): G1, negative control, received only water and feed; G2, control, received by gavage extract of *A. occidentale*; G3, control, received by gavage extract of *A. microcarpum*; G4, Paracetamol® in concentration 500 mg/kg; G5, Paracetamol® 500 mg/kg + extract of *A. occidentale* at concentration 400 mg/kg; and G6, Paracetamol® 500 mg/kg + extract of *A. microcarpum* in concentration 400 mg/kg. The animals received daily 100 *μ*L of a solution containing distilled water or extract from the leaves of *A. occidentale* or *A. microcarpum*, for a total of 30 days [31, 32]. The groups that were injured with Paracetamol® received 0.1 mL of the medication at a concentration of 500 mg/kg (Tylenol) for 8 consecutive days through gavage [33] and, on a ninth day, treatment with the crude extracts of leaves of *A. occidentale* and *A. microcarpum*.

The animals were euthanized by inhalation with 5% isoflurane. Blood obtained by retro-orbital collection (10 *μ*L) was placed on slides immediately producing the blood smear and into microtubes (2 mL) for biochemical tests. The cervical spine was displaced, and after total exsanguination, the liver was divided into two parts. One part was stored in liquid nitrogen (−196°C) and subsequently stored in the ultrafreezer at −80°C, and the other portion of the organ was immersed in Carson Formalin.

#### 2.4.2. Biochemical Analysis of Serum

The blood was centrifuged at 870 g for 10 minutes, and the serum was used for the analysis of biochemical parameters. The parameters evaluated were ALT (alanine aminotransferase), AST (aspartate aminotransferase), GGT (gamma-glutamyl transferase), ALP (alkaline phosphatase), total cholesterol, and triglycerides. The parameters were analyzed using the appropriate enzymatic colorimetric kits following the manufacturer's instructions (Assay Kit, Bioclin, Quibasa, Belo Horizonte, MG, Brazil). The serum was read by specific colorimetric assays (Bioclin®, Brazil) using a clinical chemistry analyzer BS-200 (Mindray®).

#### 2.4.3. Hematologic Analysis: Differential Leukocyte Count

Blood smears were performed with 10 *μ*L of blood collected on the day of euthanasia on histological slides and stained with the Panótico system (Laborclin Ltda.) [34]. On each slide, the fields with the lowest cell concentration were chosen for counting the differential leukocytes (neutrophil, lymphocyte, monocyte, eosinophil, and basophil), totalizing an absolute value of 100 cells. The leukocyte differential count used as a reference value was based on the values proposed by Santos et al. [35].

#### 2.4.4. Oxidative Markers

One hundred and fifty (150 mg) liver fragments were prepared for oxidative analysis. They were homogenized in 0.1 M phosphate buffer, pH 7.4, and centrifuged at 4°C. Peroxidation markers were analyzed in the supernatant (homogenate). The homogenate was mixed with thiobarbituric acid, and the formation of the thiobarbituric acid reactive species (TBARS) was monitored at 535 nm, according to the protocol previously described by Winterbourn et al. [36]. The assay of the protein carbonyl was performed using the method based on the carbonylation reaction of proteins with 2,4-dinitrophenylhydrazine (DNPH) forming dinitrophenylhydrazone [37]. The content of carbonylated proteins was calculated using the molar extinction coefficient (21 × 1031 mol cm). The results of TBARS and total proteins were normalized according to the total protein levels in the supernatant and expressed in (nmol/mg) protein [38].

#### 2.4.5. Antioxidant Enzymes

The antioxidant activity was carried out with liver samples of 150 mg, and the activity of superoxide dismutase (SOD) was estimated by the xanthine oxidase method based on the production of H_2_O_2_ and the reduction of nitroblue tetrazolium [39]. Catalase activity (CAT) was evaluated according to the protocol described by Aebi [40] by measuring the rate of decomposition of H_2_O_2_. The determination of S-transferase glutathione activity (GST) was performed on the decay rate of NADPH as described by Flohé and Günzler [41]. The total protein dosage in the homogenates was performed by the method of Lowry et al. [38], the data were normalized according to the total protein levels in the supernatant, and the results were expressed in U CAT/SOD/GST mg of protein.

#### 2.4.6. Histopathological Analysis

Liver fragments were removed and fixed for 24 hours in Carson's formalin [42] at room temperature. After fixation, the tissues were dehydrated in an increasing ethanol gradient and included in (hydroxyethyl)methacrylate-based resin (Historesin ®, Leica). Cross-sectional and longitudinal sections of 5 *μ*m thickness were obtained with the Multicut 2045 rotary microtome (Reichert-Jung, Germany) and stained with hematoxylin and eosin (HE) for analysis of cells and vessels. The cuts were then mounted in Entellan (Merck, Frankfurt, Germany). To prevent repeated analysis of the semiserial sections, only 1 every 5 sections made was used. The slides were observed, and the images were captured using light-field microscopy (Leica DM 750) connected to a digital camera (Olympus QColor-3, Tokyo, Japan). The increase chosen was 20x, in 10 fields totaling 60 fields per group.

### 2.5. Statistical Processing

Statistical programs SPSS 20 and Graph Pad Prism 5® were used. For the normality tests, Shapiro–Wilk tests were used and comparisons were made between three independent variables by analysis of variance (ANOVA). For data with normal distribution, the post hoc Tukey test was used to detect the differences between the groups that did not receive Paracetamol® (G1; G2; G3) and the ones that received Paracetamol® (G4; G5 and G6). It was considered statistically significant when *p* < 0.05. The absorbances of gallic acid and rutin solutions were plotted on a graph to produce a calibration curve, in the quantification of total phenolics and flavonoids, respectively.

## 3. Results

### 3.1. Chemical Analysis

#### 3.1.1. Quantification of Total Phenolics and Total Flavonoids

The content of phenolic compounds was 2.32 mg EAG g^−1^ and 1.64 mg EAG g^−1^ of the extract the *A. occidentale* and *A. microcarpum*, respectively. The concentrations found in 1 g of a sample of flavonoid compounds were 0.29 mg ER g^−1^ and 0.34 mg ER g^−1^ in the *A. occidentale* and *A. microcarpum* extracts, respectively.

#### 3.1.2. HPLC Analysis

**Figure 1:**
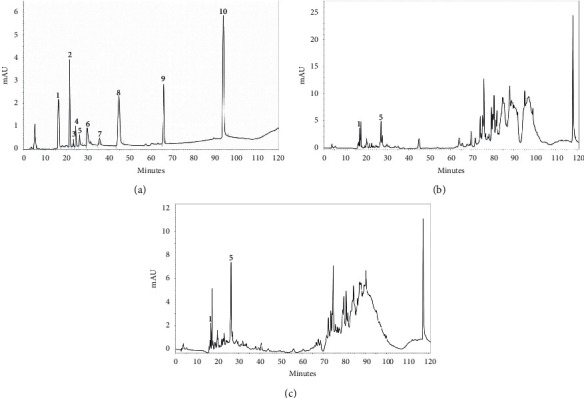
HPLC chromatogram detected at 280 nm of (a) mixture of authentic compounds of gallic acid, chlorogenic acid, vanillic acid, syringic acid, catechin, quercetin, coumaric acid, rutin, naringin, and flavone; (b) *A. occidentale* extract; and (c) *A. microcarpum* extract. Peak 1, gallic acid (Rt 16.25 min), and peak 5, catechin (Rt 26.24 min).

HPLC-UV chromatograms of mixture from authentic compounds, such as gallic acid (1; Rt 16. 25 min), chlorogenic acid (2; Rt 21.55 min), vanillic acid (3; Rt 23.35 min), syringic acid (4; Rt 24.39 min), catechin (5; Rt 26.24 min), quercetin (6; Rt 31.00 min), coumaric acid (7; Rt 35.62 min), rutin (8; Rt 44.73 min), naringin (9; Rt 65.77 min), and flavone (10; Rt 93.88 min), are represented in Figure 1(a). The HPLC fingerprint of the extracts of *A. occidentale* and *A. microcarpum* is shown in Figures 1(b) and 1(c), respectively.

By comparing the retention time of the peaks of authentic samples of phenolic compounds with the peaks of the chromatograms of the extracts, it was possible to identify the compounds gallic acid and catechin in both extracts. The two compounds were also identified in the extracts by coinjection of these standards. The detection at the wavelength at 280 nm showed the best selectivity for the detection of phenolic compounds in the extracts.

### 3.2. In Vivo Study

#### 3.2.1. Biochemical Analyses on Serum Concentration in IL-10 Knockout Mice

The mice of the group that received Paracetamol® and the extracts of *A. occidentale* (G5) presented a reduction of ALT levels when compared to the group treated only with Paracetamol® (G4). The mice treated with extract of *A. occidentale* (G2) and *A. microcarpum* (G3) presented a reduction of AFO when compared to the control group (G1), and the (G5 and G6) treated groups showed too reduction when compared to the control group (G1). The animals receiving *A. microcarpum* and *A. occidentale* extract (G2 and G3) showed a reduction in serum TAG values when compared to control animals (G1), and *A. occidentale*-treated mice reduced TAG levels compared to control (G1) (Table 1).

#### 3.2.2. Differential Leukocyte Count

**Figure 2:**
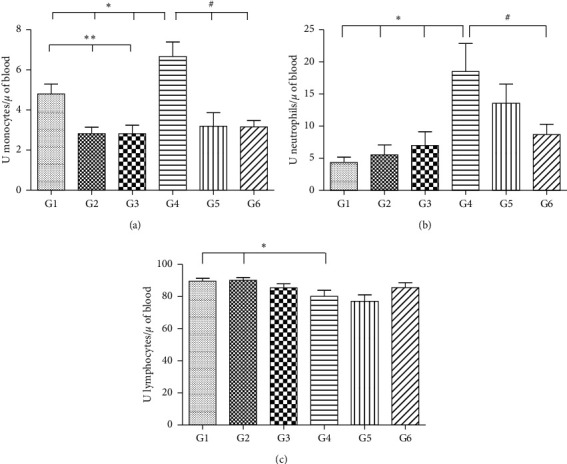
The number of monocytes (a), neutrophils (b), and lymphocytes (c) per microliter of blood in 100 cells counted in IL-10 knockout mice (^*∗*^significant differences between control and paracetamol groups; ^*∗∗*^significant differences between control groups; ^#^significant differences between treated groups, *p* < 0.05). One-way ANOVA statistical test and Tukey test. G1, control; G2, *A. occidentale*; G3, *A. microcarpum*; G4, Paracetamol®; G5, Paracetamol® + *A. occidentale*; G6, Paracetamol® + *A. microcarpum*.

The number of monocytes present in the control group (G1) was greater than that in the groups receiving the extracts of *A. occidentale* (G2) and *A. microcarpum* (G3). This result was expected since IL-10 knockout animals naturally have a larger quantity of inflammatory cells. On the other hand, animals that received Paracetamol® only (G4) also presented increased levels of these cells compared to the groups receiving Paracetamol® associated with *A. occidentale* (G5) and *A. microcarpum* (G6). The group that received paracetamol (G4) showed a significant increase in monocytes compared to controls. The number of neutrophils increased in the group that received paracetamol (G4) compared to controls (G1, G2, and G3), and concerning lymphocytes, there was a reduction in the number of these cells in the G4 group when compared to G1 and G2 (Figure 2). Basophils and eosinophils had an average that was between 0 and 1, in 100 cells, and did not show significant differences between groups, and no young cells were found.

#### 3.2.3. Quantification of Oxidative Stress Markers in Liver Samples

**Figure 3:**
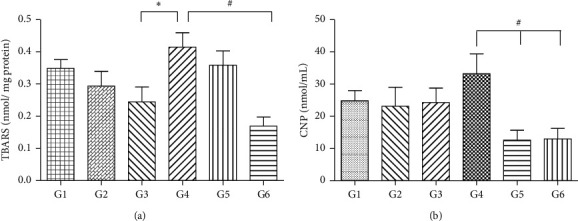
Contents of TBARS (a) and carbonylated protein (CNP) (b) in the liver tissue of IL-10 knockout mice (^*∗*^significant differences between control and paracetamol groups; ^#^significant differences between treated groups, *p* < 0.05). One-way ANOVA statistical test and Tukey test. G1, control; G2, *A. occidentale*; G3, *A. microcarpum*; G4, Paracetamol®; G5, Paracetamol® + *A. occidentale*; G6, Paracetamol® + *A. microcarpum*.

The group treated with *A. microcarpum* (G6) extract associated with Paracetamol® presented a decrease in TBARS levels compared to the group that received only Paracetamol® (G4), and the group that received paracetamol (G4) showed an increase concerning the control (G3) (Figure 3(a)). The content of carbonyl proteins (CNP) was lower in the group treated with extracts of *A. occidentale* (G5) and *A. microcarpum* (G6) when compared to the group that received only Paracetamol® (G4) (Figure 3(b)).

#### 3.2.4. Evaluation of Antioxidant Enzymes in the Liver

**Figure 4:**
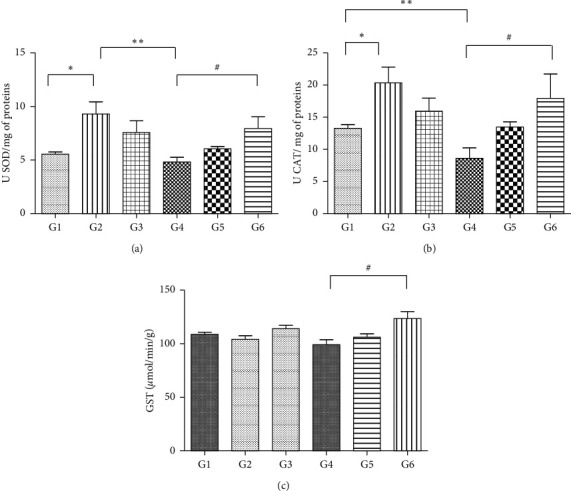
The activity of the antioxidant enzymes ((a) SOD; (b) CAT; (c) GST) in liver maceration of IL-10 knockout mice (^*∗*^significant differences between control groups; ^*∗∗*^significant differences between control and paracetamol groups; ^#^significant differences between treated groups, *p* < 0.05). One-way ANOVA statistical test and Tukey test. G1, control; G2, *A. occidentale*; G3, *A. microcarpum*; G4, Paracetamol®; G5, Paracetamol® + *A. occidentale*; G6, Paracetamol® + *A. microcarpum*.

There was an increase in superoxide dismutase (SOD) and catalase (CAT) enzymes (G2) compared to the negative control (G1). CAT showed a reduction in the group that received Paracetamol® (G4) compared to the control (G1) and SOD compared to the control that received *A. occidentale* (G2). The group that was treated with *A. microcarpum* (G6) increased its activity in relation to the treatment with paracetamol (G4). In the analyzes of glutathione peroxidase (GST), there was an increase in the group treated with *A. microcarpum* in relation to the group that received Paracetamol® (Figure 4(c)).

#### 3.2.5. Histopathological Analyses

**Figure 5:**
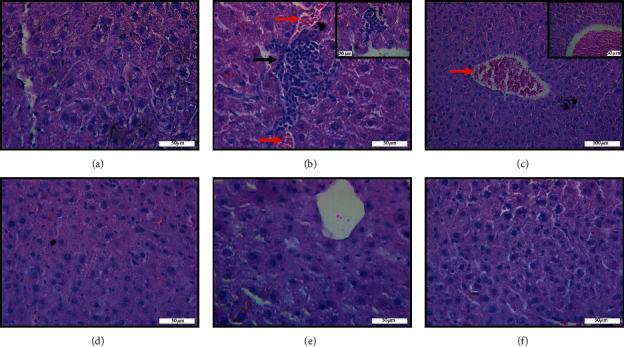
Representative photomicrographs of histological sections of the liver of IL-10 knockout mice stained with hematoxylin and eosin submitted to injury by Paracetamol® ((a) control; (b) Paracetamol®, injury magnification (100x); and (c) Paracetamol®, injury magnification (40x); (d) and (e) Paracetamol® + *A. occidentale*; (f) Paracetamol® + *A. microcarpum*) observed under the light microscope. Black arrows indicate inflammatory infiltrate, and red arrows indicate vascular congestion. Bar 20 *μ*m = 100x, bar 50 *μ*m = 40x ,and bar 100 *μ*m = 20x.

The animals treated with *A. occidentale* (G5) showed a reduction in the inflammatory infiltrate, nuclear volume, sinusoid congestion, and cholestasis, and those who received *A. microcarpum* (G6) differed only from sinusoidal congestion (Figure 5; Table 2). The group Paracetamol® (G4) was effective in inducing infiltrate, increased nuclear volume, sinusoidal congestion, and hepatitis cholestasis compared to controls (G1, G2, and G3). The controls that received both extracts (G2 and G3) showed a reduction in nuclear volume, with the negative control (G1) and the inflammatory infiltrate only in the group that received *A. microcarpum* (Table 2).

## 4. Discussion

The present study investigated the effect of *A. occidentale* and *A. microcarpum* extracts on the liver injury caused by paracetamol on IL-10 knockout mice. The experimental injury of paracetamol was previously validated as a model of oxidative and inflammatory liver injury [43, 44]. Toxicity by paracetamol occurs due to the formation of peroxynitrite and an increase of nitric oxide responsible by degeneration of cytoplasmic and nuclear fragmentation by endonucleases. In addition, these compounds promote hepatic injury due to an increase in the number of inflammatory cells with the release of mediators leading to inflammation, degeneration, and finally necrosis. In this sense, in order to show the positive effects of the extracts on liver damage caused by the hepatotoxic agent (paracetamol), a dose is known to cause acute and severe damage to the liver was used, that is, a megadose of 500 mg/kg of the animal. Besides, in a similar study when the 300 mg/kg dose was used in CD-18-deficient mice, acute hepatoxicity was observed with the opening of the mitochondrial membrane potential leading to death cells compromising all the hepatic functions [43]. Using the wild-type strain, the acetaminophen induced in experimental albino rats shows significant elevation in levels of the serum marker enzymes, aspartate transaminase and alanine transaminase, and of lipid peroxides with decreased levels of antioxidant enzymes such as superoxide dismutase and catalase, showing its high harmful potential [45]. In addition, this protocol has also been widely used for analyzing the protective and curative effects of plant extracts, especially associated with pathologies of the digestive system [46, 47].

The prevalence of liver diseases induced by chemical agents has increased progressively over recent decades in various countries [48]. The liver is involved in xenobiotic metabolism, playing an important role in detoxifying the organism. Following exposure to hepatotoxic compounds such as products originating from fungi, bacterial metabolites, heavy metals, environmental pollutants, and chemotherapeutic agents, the liver becomes vulnerable to several disorders [10, 49]. Biodiversity is a great reservoir of bioactive secondary metabolites that, in conjunction with traditional knowledge, has led to the discovery of drugs for the treatment of different human pathologies. In this context, various countries have sought to introduce medicinal plants as an integrated and supplementary practice in primary health care. However, although different plants are used in popular medicine, it is estimated that <10% of plants have been investigated in sufficient depth to evaluate their therapeutic properties [16]. Therefore, *A. occidentale* and *A. microcarpum* extracts used in our study already are used in Brazilian traditional medicine for the treatment of infections, throat inflammation, bronchitis, arthritis, intestinal colic, jaundice, diabetes, and asthma [50]. In addition, in a previous study that analyzed the toxicity of *A. occidentale* was observed that the dose of 400 mg/kg did not present toxic activity, and on the contrary, this did promote tissue repair and antioxidant protection [51, 52].

Detecting the compounds of secondary metabolism of plants provides a better understanding of their metabolic activities, permitting that their physiological mechanisms be inferred [1, 3, 4]. High levels of phenolic compounds, mainly flavonoids and tannins, may be responsible for the antioxidant activity of the *Anacardium* species because several reports are attributing this property to these groups of secondary metabolites [53, 54]. The HPLC fingerprint chromatograms show the presence of gallic acid (peak 1) and catechin (peak 5), with retention times coinciding with the authentic compounds. Gallic acid and catechin are precursors to hydrolyzable and condensed tannins, respectively, common in the tissues of the Anacardiaceae family, mainly in the secretory and mesophilic epithelium, which suggests the presence of these metabolites in the leaves of the two extracts [53]. In addition, our study corroborated with Salehi et al. [4] that detected polyphenolic compounds in leaves of *Anacardium* species as tannins coumarins, and saponins, compounds that can activate antioxidant and anti-inflammatory capacity. Also, in other studies but using *in vitro* models, the authors demonstrated the presence of phenolic compounds and antioxidant activities in *Anacardium* leaf extracts showing free radical scavenging activity, ferric reducing power, and ferrous ion chelating ability, reinforcing the plant's antioxidant activity [55]. Corroborating these findings, Filho et al. [2] showed that *A. microcarpum* stem bark extract has gallic acid and catechin, and these compounds promoted a reduction in the lipid peroxidation reinforcing the antioxidant activity of these compounds.

In general, the biochemical analyses showed that there was no hepatic impairment in the mice that received only the extracts, which is a strong indicator of the absence of toxicity of the extract. The group that received Paracetamol® had increased levels of the liver enzyme ALT (alanine aminotransferase) when compared to the groups that received Paracetamol® and were treated with *A. occidentale* (cashew). These biochemical enzymes that evaluate hepatic metabolism are indirect indicators of liver and bile duct alteration, so it is important to perform the dosage after treatment with an herbal remedy. ALT activity is an important biochemical parameter for the detection of hepatotoxicity, and other markers such as AST are complementary to the result [56, 57]. The combination of these markers provides more accurate results in the presence of liver damage. GGT and alkaline phosphatase are also considered important additional markers of liver function, including a differential diagnosis of biliary function [18]. The data indicate that the extracts may act as protectors of liver function since even if the animals did not reach the reference values for these parameters, there was still a significant decrease in their values. Tédong et al. [58] evaluated the liver of mice after receiving leaf extract from *A. occidentale* and observed that up to 14 g of extract per kg of the animal via gavage is nontoxic. The hepatoprotective index of a drug can be evaluated by its ability to reduce harmful effects and preserve liver morphophysiology. Therefore, AST, ALT, and GGT are often used as indirect markers of hepatic injury and are considered sensitive parameters to evaluate functional status [44, 59]. Triglycerides showed a significant reduction between the control groups and those that received *A. occidentale* and *A. microcarpum* extracts, suggesting that the extracts may act as a hypolipidemic, decreasing the levels of plasma triglycerides. This result calls our attention to, in the future, assessing the ability of extracts to prevent the formation of atheroma in a specific cardiovascular disease (CVD) model.

Free radicals promote changes in cellular membranes and consequently also in the redox balance of the cell, leading to degenerative processes associated with inflammation [57]. The regulation of the inflammatory process occurs from the release of proinflammatory mediators, which may be associated with the expression of NF-kB that regulates genes responsible for the generation of mediators or proteins in inflammatory processes. It is found in the cytoplasm linked to regulatory proteins I Bs, in response to different stimuli, such as infection, hypoxia by oxidative stress, extracellular signals, and inflammation, and the regulatory proteins I Bs are phosphorylated by the enzyme kinase I B. The activation of NF-kB regulates inflammatory proteins and TNF and IL-1 [60]. Such changes were observed in our results when the groups were treated exclusively with Paracetamol®, and there was an increase in lipid and protein peroxidation and protein oxidation markers in the hepatic tissue. Lipid peroxidation and protein oxidation are typical metabolic processes related to the pathogenesis of morphofunctional liver injuries [61, 62]. Although lipid peroxidation also occurs under physiological conditions, external factors may amplify this process, leading to intense membrane lipid oxidation and eventually cell death [63, 64]. Generally, these markers reflect the level of stress caused by the release of free radicals *in vivo*, because hydroperoxides are one of the main products of the decomposition of polyunsaturated fatty acids from the plasma membrane during oxidative events [58, 64]. In the present study, there was a decrease in these markers after the use of extracts of *A. occidentale* and *A. microcarpum*, with the induction of stress by Paracetamol®, which suggests a protective effect of these extracts during the liver intoxication process. Similar results were reported by Filho et al. [2] using a treatment in brains of mice with fractions of extracts of *A. microcarpum* bark. A significant reduction was observed in lipid peroxidation when testing the ethanolic fraction. On the other hand, Broinizi et al. [32] tested cashew peduncles (*A. occidentale*) in the hepatic tissue of Wistar rats and demonstrated that there were no significant differences for the determination of lipid peroxidation by TBARS amongst the treatment groups. These findings show that new studies analyzing other oxidative/nitrosative markers are necessary and that the results presented in this study must be interpreted with caution, once that the methodologies used and the evaluation parameters are extremely heterogenic, with different measures being reported in all comparative studies.

In the processes of biological oxidation, CAT and SOD play an important role in protecting the liver against the toxic effects of many xenobiotics [65], representing a cellular defense mechanism against reactive oxygen species. In our study, we observed that the groups that received only the extract of *A. occidentale* and the group that received an extract of *A. microcarpum* associated with Paracetamol® presented an increase of the antioxidant enzymes SOD and CAT. These findings demonstrate that these extracts acted like an antioxidant system defending the liver tissue against reactive oxygen species (ROS), resulting in the reduction of oxidative damage. SOD and CAT are the first enzymes involved in the antioxidant defense process of the cell and therefore are extremely important markers in the study of intracellular antioxidant systems [66, 67]. Another interesting point observed in our study was that the groups that received only paracetamol presented a reduction in the number of these antioxidant enzymes. Similar to our findings, studies showed that during intense tissue oxidation, levels of antioxidant enzymes are often reduced due to the high levels of production and accumulation of O_2_ induced by xenobiotics that increase enzyme consumption in intoxicated tissues [65, 68]. In the analysis of glutathione S-transferase, the mice that received treatment with the extract of *A. microcarpum* + Paracetamol® showed an increase in the activity of these enzymes when compared to the group that received only paracetamol.

These findings demonstrate the efficacy of *A. microcarpum* in the generation of the second line of defense of antioxidant systems. Results similar to those described in our study were found by Ukwenya et al. [69] who analyzed the effect of *A. occidentale* leaves extract on glucose-6-phosphate-dehydrogenase (G6PDH), GST, and SOD activities and demonstrated potent antioxidant effects of these extracts in the stimulation of these antioxidant enzymes, as well as, increased G6PDH, inhibiting lipid peroxidation.

In the present study, monocyte and neutrophil quantity showed an increase in the number of these cells, and lymphopenia in the control group (G1) and paracetamol group (G4), when compared to the groups that received the two extracts. This was an expected result since IL-10 knockout animals naturally present more inflammatory cells. Another interesting fact that demonstrates the inflammatory condition presented by this knockout model is that the amount of inflammatory infiltrate in the hepatic tissue was also higher. Performing a differential count of leukocytes and identifying the main cell types can help elucidate the pathological process and identify the main mechanisms involved in the protection exerted by the proposed extracts [35]. On the other hand, animals that received only Paracetamol® also had increased levels of these cells compared to the groups receiving Paracetamol® associated with *A. occidentale* and *A. microcarpum* extracts. These findings demonstrate the harming power of Paracetamol® on hepatic tissue and, at the same time, demonstrate the significant protective role of the extracts on inhibiting the inflammation. Taking into consideration that, in general, the damaging mechanisms go through the development of an inflammatory process, where mediators that can cause important cellular and vascular changes in the tissues are released [70], developing a therapy that inhibits or diminishes tissue inflammation is highly desirable.

The histopathological analysis represents a reproducible and reliable method used to evaluate the hepatoprotective effect of a drug, especially in cases in which the chemical characteristics and dose of chemical compounds provide sufficient toxicity to induce morphological tissue disorganization [71]. Therefore, to check for possible hepatocellular damage in the mice, the histopathological study was done, complementing the data obtained in the enzymatic and biochemical analyses. In our study, an increase of the inflammatory infiltrate was observed after exposure to paracetamol, possibly associated with the knockout model. In addition, there was an increase in nuclear volume, vascular congestion, and cholestasis in the groups that received Paracetamol® mainly compared to the groups that received Paracetamol® associated with the extracts. Based on the data, it is believed that the extracts have high anti-inflammatory power, probably due to their rich polyphenols. Extracts rich in these compounds are known to decrease venous stasis by improving cell migration and tissue oxygenation [72–74]. Similar results were obtained by Wang et al. [75] after treatment with *Glechoma hederacea* extract at the dose of 0.5 g/kg for 4 weeks demonstrated an effect in the reduction of cholestatic liver injury. The extract of *A. occidentale* and *A. microcarpum* showed a possible decrease in this process, since after the treatment, the tissue presented very similar characteristics to the normal tissue. All these findings suggest that the extracts produced no toxicity at the dose tested and showed hepatoprotective effects.

## 5. Conclusions

The present study showed that extracts from the leaves of *A. microcarpum* and *A. occidentale* exerted a hepatoprotective effect against lesions caused by Paracetamol®, in IL-10 knockout mice. The results indicated that both extracts are functional foods with antioxidant activity, as they decreased the production of oxidative markers and increased the production of antioxidant enzymes. In addition, there was a decrease in the inflammatory infiltrate and in the number of monocytes in the groups that were treated with the extracts after receiving Paracetamol®. Further pharmacological evaluations are still essential to identify the active components of this extract and to fully clarify its action mechanisms, which may be associated with high potential for the prevention and treatment of acute and chronic liver injuries.

## Figures and Tables

**Table 1:** Effects of treatment with *A. occidentale* and *A. microcarpum* on liver function tests of IL-10 knockout mice (*n* = 36).

mg/dl	G1	G2	G3	G4	G5	G6
ALT	77.83 ± 16.74	69.50 ± 14.12	71.00 ± 12.17	72.60 ± 6.98	^*∗*^58.00 ± 4.00	70.80 ± 6.38
AST	208.20 ± 12.56	191.3 ± 24.70	186.20 ± 36.16	209.4 ± 79.84	140.20 ± 65.00	176.6.2 ± 19.50
AFO	^*∗*#^164.40 ± 49.93	^*∗*^87.50 ± 30.29	^*∗*^66.67 ± 18.93	104.40 ± 32.97	^#^84.00 ± 26.68	^#^98.50 ± 19.49
GGT	35.40 ± 7.47	48.00 ± 7.58	47.50 ± 6.12	39.80 ± 16.38	34.00 ± 12.08	41.00 ± 14.75
TC	110.83 ± 13.69	107.20 ± 20.00	107.14 ± 23.29	98.33 ± 17.82	85.00 ± 13.84	104.83 ± 8.61
TAG	^*∗*#^180.33 ± 23.81	^*∗*^121.33 ± 42.42	^*∗*^110.80 ± 28.17	91.40 ± 11.48	^#^93.60 ± 31.70	123.60 ± 17.17

ALT, alanine aminotransferase; AST, aspartate aminotransferase; AFO, alkaline phosphatase; GGT, gamma-glutamyl transferase; TC, total cholesterol; TAG, triglycerides; one-way ANOVA and Tukey statistical tests (G1, control; G2, *A. occidentale*; G3, *A. microcarpum*; G4, Paracetamol®; G5, Paracetamol® + *A. occidentale*; G6, Paracetamol® + *A. microcarpum*). ^*∗*^Significant differences between control groups; ^#^ significant differences between treated groups, *p* < 0.05.

**Table 2:** Effects of treatment with *A. occidentale* and *A. microcarpum* on histopathological analysis of the livers of IL-10 knockout mice treated with Paracetamol® and *A. occidentale* and *A. microcarpum* extracts.

	G1	G2	G3	G4	G5	G6
Infl. infil.	6.60 ± 0.89^a,c^	5.00 ± 1.58^a^	4.16 ± 1.47^a,c^	7.50 ± 1.04^a,b^	4.33 ± 0.51^b^	4.50 ± 2.16^b^
Nucl. vol.	6.50 ± 1.12^a,c^	3.30 ± 1.03^a,c^	2.00 ± 0.89^a,c^	8.50 ± 1.04^a,b^	2.80 ± 0.98^b^	2.00 ± 0.63^b^
Sin. cong.	6.16 ± 1.94^a^	6.30 ± 1.03^a^	7.30 ± 1.50^a^	8.70 ± 1.36^a^	4.16 ± 2.78^b^	5.50 ± 2.07
Colest.	1.83 ± 1.16^a^	1.00 ± 1.26^a^	1.50 ± 0.83^a^	6.83 ± 0.98^a,b^	0.66 ± 0.51^b^	1.16 ± 1.60^b^

Infl. infil., inflammatory infiltrate; Nucl. vol., nuclear volume; Sin. cong., sinusoidal congestion; Colest., cholestasis; different letters in columns denote statistic differences between the groups (*p* < 0.05).One-way ANOVA statistical test and Tukey test. G1, control; G2, *A. occidentale*; G3, *A. microcarpum*; G4, Paracetamol®; G5, Paracetamol® + *A. occidentale*; G6, Paracetamol® + *A. microcarpum*.

## Data Availability

The data used to support the findings of this study are included within the manuscript.
